# Cognitive Protective Mechanism of Crocin Pretreatment in Rat Submitted to Acute High-Altitude Hypoxia Exposure

**DOI:** 10.1155/2020/3409679

**Published:** 2020-06-09

**Authors:** Xiaoyan Zhang, Xianjun Zhang, Zhancui Dang, Shanshan Su, Zhanqiang Li, Dianxiang Lu

**Affiliations:** ^1^Research Center for High Altitude Medicine, Key Laboratory of High Altitude Medicine, Ministry of Education, Qinghai University, Xining 810001, China; ^2^Key Laboratory of Application and Foundation for High Altitude Medicine Research in Qinghai Province, Xining 810001, China; ^3^Department of Basic Medicine Science, Medical College, Qinghai University, Xining 810001, China; ^4^Technical Center of Xining Customs District, Key Laboratory of Food Safety Research in Qinghai Province, Xining 810003, China

## Abstract

Inadequate oxygen availability at high altitude leads to oxidative stress, resulting in hippocampal neurodegeneration and memory impairment. In our previous study, we found that the cognitive dysfunction occurred when male SD rat was rapidly exposed to 4200 m of high altitude for 3 days. And we also found that crocin showed a cognitive protective effect under hypoxia by regulating SIRT1/PGC-1*α* pathways in rat's hippocampus. In this article, focused on factors related to SIRT1/PGC-1*α* pathways, we proposed to further elucidate crocin's pharmacological mechanism. Adult male Sprague-Dawley rats were randomly divided into five groups: control group, hypoxia group (rats were rapidly transported to high altitude of 4200 m for 72 h), and crocins+hypoxia groups (pretreatment with crocin of 25, 50, and 100 mg/kg/d for 3 days). The learning and memory ability was tested by Morris water maze analysis. Hippocampal histopathological changes were observed by HE staining and Nissl staining. The expression of NRF1, TFAM, Bcl-2, Bax, and caspase-3 was detected by immunohistochemistry, RT-PCR, and western blotting test. The contents of malondialdehyde (MDA), superoxide dismutase (SOD), glutathione (GSH), and glutathione peroxidase (GSHPx) were detected by the TBA, WST, and colorimetry method. Neuronal apoptosis was observed by TUNEL staining. After crocin pretreatment, the traveled distance was significantly reduced and the percentage of time in the target quadrant was significantly increased tested by Morris water maze. And neuronal damage in the hippocampus was also significantly ameliorated based on HE staining and Nissl staining. Furthermore, in hippocampus tissue, mitochondrial biosynthesis-related factors of NRF1, TFAM expression was increased; oxidative stress factors of SOD, GSH, and GSHPx expression level were increased, and MDA and glutathione disulfide (GSSG) level were decreased; antiapoptotic protein Bcl-2 expression was increased, and proapoptotic proteins Bax and caspase-3 expression were decreased, with a manner of crocin dose dependent. Therefore, the cognitive protective mechanism of crocin in rat under acute hypoxia was related to promoting mitochondrial biosynthesis, ameliorating oxidative stress injury, and decreasing neuronal apoptosis.

## 1. Introduction

In the high-altitude area, the atmospheric pressure drops and the oxygen content is low, which causes a decrease in the partial pressure of oxygen at every point along the oxygen transport cascade from ambient air to cellular mitochondria. Acute high-altitude hypoxia affects the blood flow and its consequent distribution to organs and the efficiency of O_2_ utilization. And the brain, which is the most susceptible organ to hypoxic damage due to its high energy demands, is very vulnerable to hypoxia which could cause headaches, dizziness, blurred vision, tinnitus, spatial learning and memory impairment, and even pathological changes [1–3]. Additionally, high-altitude hypoxia affects severely the structural integrity of the principal neurons in the hippocampus, resulting in decreased hippocampus-dependent learning and memory function [4, 5]. The pathological features of hippocampus injury induced by acute hypobaric hypoxia include mitochondrial dysfunction, morphological changes, and upregulation of genes associated with apoptosis [6]. In addition, hypoxia also induces an imbalance between free radical generation and antioxidant protection, resulting in an oxidative damage of biomolecules [7, 8]. Therefore, it is of utmost importance to provide protective measures against brain damage due to acute hypoxia. The treatment or improvement of central nervous system damage due to acute high-altitude hypoxia has recently attracted increasing attention in the field of high-altitude medicine [9, 10]. SIRT1 (silent information regulatory factor 1) deacetylation of PGC-1*α* (peroxisome proliferator-activated receptor gamma coactivator-1*α*) has been extensively implicated in mitochondrial biogenesis, oxidation stress, and apoptosis [11, 12]. Studies showed that upregulating the expressions of SIRT1 and PGC-1*α* level could preserve mitochondrial function and attenuate oxidative stress injury [13] by increasing NRF1 (nuclear respiratory factor 1) and TFAM (mitochondrial transcription factor A) protein expressions, enhancing SOD activities and suppressing the contents of ROS and MDA [14].

The drugs currently used to prevent acute altitude sickness include acetazolamide, dexamethasone, montelukast, and aspirin, but these drugs have varying degrees of side effects, including sensory abnormalities, gastrointestinal bleeding, osteoporosis, and increased risk of infection [15]. Plants are excellent sources of bioactive compounds throughout history in the search for new drugs. Crocin, a water-soluble carotenoid, is regarded as the quality marker for the quality control of saffron, which has been extensively used for the treatment of brain and cardiovascular diseases in traditional Tibetan medicine (Chinese Pharmacopoeia Commission, 2015, Figure 1). Crocin showed the effects of antiapoptosis, antioxidation, neuroprotection, and inhibition of mitochondrial dysfunction [16–18]. Our previous study found that crocin pretreatment improved learning and memory in rat under acute high-altitude hypoxia, and it could upregulate the level of SIRT1 and PGC-1*α* in the hippocampus [19]. But the further cognitive protective mechanism was poorly understood.

In this article, we focused on key factors regulated by the SIRT1/PGC-1*α* pathway, mainly concerned with mitochondrial biosynthesis (NRF1 and TFAM), oxidation stress (SOD, GSH, GSSG, GSHPx, and MDA), and apoptosis (Bax, Bcl-2, and caspase-3), to further illustrate the underling brain protective mechanism of crocin under acute hypoxia exposure.

## 2. Materials and Methods

### 2.1. Medicine and Reagents

**Figure 1:**
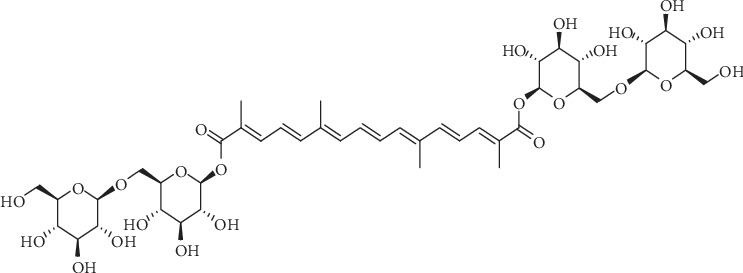
Chemical structure of crocin.

Crocin was purchased from Sigma-Aldrich (purity above 98%; Sigma-Aldrich, Japan; catalog #BCBV9507) (Figure 1) and dissolved in normal saline. Hematoxylin-eosin (catalog #AR1180-100), rabbit anti-TFAM (antibody catalog #PB0413), and BCA Protein Assay Kit (catalog #AR1189) were purchased from Boster Biological Technology (Wuhan, China). Trizol reagent (catalog #15596-026) was purchased from Invitrogen; Nissl solution (catalog #C0117) was purchased from Beyotime Biotechnology (Beijing, China). Rabbit anti-NRF1 (antibody catalog #ab175932), rabbit anti-Bcl-2 (antibody catalog #ab194583), rabbit anti-Bax (antibody catalog #ab53154), rabbit anti-caspase-3 (antibody catalog #ab4051), and rabbit anti-GAPDH (antibody catalog #ab9485) antibodies were supplied from Abcam Biotechnology (Cambridge, MA, USA). Goat anti-rabbit IgG-HRP (catalog #BA1056) were purchased from Boster Biological Technology (Wuhan, China). SOD (catalog #A001-3-2), GSH (catalog #A006-2-1), GSHPx (catalog #A005-1-2), MDA (catalog #A003-1-2), and GSSG (catalog #A061-1-1) commercial kits were obtained from Jiancheng Biological Technology (Nanjing, China). Takara PrimeScript RT reagent kit and Takara TB Green™ Premix Ex Taq™ (Tli RNaseH Plus) kit were obtained from Takara Biotechnology Co., Ltd (RR820A, Dalian, China).

### 2.2. Animals

Adult male SD rats (210–230 g) of specific pathogen free (SPF) were obtained from the Experimental Animal Center of Xi'an Jiaotong University with a certificate number (license No. SCXK 2017-003). All experimental protocols were approved by the Animal Experimentation Ethics Committee of Qinghai University, and appropriate efforts were applied to minimize distress and pain of the experimental animals.

Animal model experiment was based on our previous research method [19]. Briefly, rats were randomly assigned to five experimental groups (*n* = 18, each group): control group (Ctr, saline), acute high-altitude hypoxia group (AHH, saline), low dose of crocin-pretreated hypoxic group (LHG, 25 mg/kg/d), middle dose of crocin-pretreated hypoxic group (MHG, 50 mg/kg/d), and high dose of crocin-pretreated hypoxic group (HHG, 100 mg/kg/d). Crocin (dissolved in 1 mL saline) was administered 3 days before the acute hypoxia exposure by intramuscular drug once per day. The rats in the control group were treated with a corresponding volume of normal saline. Rats were fed in an environmentally controlled room at 20 ± 2°C, with free access to food and water. The rats in the acute high-altitude hypoxic group and the crocin-pretreated hypoxic group were exposed to the acute hypoxic environment at 4200 m for 3 days (the Gande County of Qinghai Province, China), where the air density was 0.802 kg/m^3^, approximately 62% of the air density at sea level. As oxygen varies in direct proportion with air, the oxygen content in the air was also 62% of that at sea level [19].

### 2.3. Morris Water Maze (MWM) Test and Physiological Index

Rats were subjected to a daily session of four training trials at intervals of 30 min for five consecutive days [20] at approximately the same time before the induction of acute high-altitude hypoxia. The maze consisted of a round black metal tank of 160 cm in diameter with a transparent circular platform placed 2 cm below the water surface and fixed in one of the four quadrants during the training. The trial was stopped once the rats reached the platform. If the rats were unable to reach the platform within 60 s, they were placed on the platform for 10 s and then removed from the pool. A video camera linked to a computer directly located above the MWM together with an imaging analysis system (Institute of Materia Medica, Chinese Academy of Medical Sciences, China) was used to record the percentage of time spent in the target quadrant, and the traveled distance of the swim path.

After hypoxia exposure for 3 days, rats were weighed and their body weights were recorded. The same group of rats were anesthetized with urethane (1.0 g/kg) and sacrificed after the behavioral MWM test. Blood samples (0.5 mL) were taken from the abdominal aorta and used for blood cell analysis using blood cell counter (BC-5000 Vet, Mindray company, Guangzhou, China). Red blood cells (RBC), platelets (PLT), and white blood cells (WBC) were measured.

One portion of the hippocampus of 6 rats in each group was fixed in 4% paraformaldehyde solution, while the other portion was fixed in 3% glutaraldehyde for further histopathological assessment and immunohistochemical test, respectively. The hippocampus of the remaining rats was flash-frozen in liquid nitrogen and stored at −80°C for western blotting, qRT-PCR, and antioxidation index analysis.

### 2.4. H&E Staining

Brain tissues were prefixed in 4% paraformaldehyde, subsequently washed in running tap water for 24 h and then subjected to routine dehydration and paraffin embedding. Brain tissues were cut into 3 *μ*m thick serial sections by Leica microtome (Leica, Germany). Brain sections were then deparaffinized in xylene, rehydrated via graded alcohols, and stained with H&E, according to a standard protocol. Pathological changes were observed under an optical microscope (Leica, Heidelberg, Germany).

### 2.5. Nissl Staining

Brain sections were dewaxed and then placed in deionized water for 1 min, transferred to a Nissl solution at 37°C for 5 min to achieve an optimal staining, and quickly washed in deionized water. Next, slides were immersed in 70% ethanol for 5 second twice and mounted on slide using neutral gum; sections were photographed by the Leica microscope. Image-Pro Plus 6.0 software (Media Cybernetics Inc., Maryland, USA) was used to analyze the image and calculate the IOD value. The IOD value of each slice = sum of three discontinuous visual field IOD value/3.

### 2.6. TBA, WST, and Colorimetry Method

The liquid nitrogen frozen complete hippocampal tissues were placed on ice and homogenized. The homogenates were centrifuged at low temperature at 13000 g for 15 min. The supernatant was collected and protein quantified, hippocampus SOD was determined by water-soluble tetrazolium-1 (WST-1), MDA was determined by thiobarbituric acid (TBA) colorimetry, GSHPx activity was based on the consumption of reduced glutathione, and GSH/GSSG content was detected by dithio-bis-nitrobenzoic acid (DTNB) (Jiancheng Bioengineering Institute Co., Ltd., Nanjing, China).

### 2.7. Immunohistochemical Staining

The whole hippocampus was cut at a thickness of approximately 3 *μ*m. Sections were deparaffinized with xylene and rehydrated with ethanol. Sections were incubated overnight at 4°C with the following primary antibodies: rabbit anti-rat, NRF1 (1 : 50), TFAM (1 : 50), Bcl-2 (1 : 200), Bax (1 : 200), and caspase-3 (0.002 mg/mL). The sections were rinsed in PBS 3 times for 5 min, incubated with the appropriate biotinylated secondary antibody followed by an avidin–biotin horseradish peroxidase complex (Boster Biological Technology, Wuhan, China), with diaminobenzidine (DAB) as substrate. All incubations were performed in a humidified chamber. The negative control sections from each animal were subjected to the same staining procedure, except that the primary or secondary antibody was omitted. Sections were photographed by the Leica microscope; three random and nonoverlapping positively stained microscopic fields at 400x magnification were examined in each section of the hippocampal CA1 region. The true color image analysis Image-Pro Plus 6.0 software (Media Cybernetics Inc., Maryland, USA) was applied to determine the integral optical density (IOD) values of protein (IOD value of each slice = sum of three discontinuous visual field IOD value/3).

### 2.8. Western Blotting

Total proteins were isolated from the frozen hippocampus. The hippocampus was homogenized in Radio Immuno Precipitation Assay (RIPA) buffer (50 mM Tris (pH 8.0), 150 mM sodium chloride, 0.1% sodium dodecyl sulphate (SDS), 0.5% sodium deoxycholate, and 1% Triton X-100) mixed with phenylmethanesulfonyl fluoride (PMSF) and then sonicated for ice cold water. The homogenates were centrifuged at 2000 g for 20 min at 4°C, and protein concentration of the supernatant was quantified using the BCA method. Adjust the protein concentration to 2 *μ*g/*μ*L for sample preparation, equal amounts of protein (20 *μ*g) were loaded in each lane of 10% SDS-polyacrylamide gels. After the electrophoresis, proteins were transferred to nitrocellulose membranes and incubated with 5% skim milk for 2 h at room temperature. Membranes were incubated overnight at 4°C with the following primary antibodies: rabbit anti-rat NRF1 (1 : 1000), rabbit anti-rat TFAM (1 : 1000), rabbit anti-rat Bcl-2 (1 : 2000), rabbit anti-rat Bax (1 : 2000), rabbit anti-rat caspase-3 (1 : 500), and rabbit anti-rat *β*-actin (1 : 2500) used as the loading control. The membrane was washed 3 times with PBS for 5 min each time and exposed to a horseradish peroxidase-conjugated goat anti-rabbit secondary antibody (1 : 5000) for 1 h at room temperature. The membrane was then developed using enhanced chemiluminescence (ECL, Santa Cruz Biotechnology Inc.), followed by autoradiography. The films were scanned, and the optical density was determined using the TANON GIS analysis system.

### 2.9. Quantitative Real-Time PCR (qRT-PCR)

Total RNA was isolated from the frozen hippocampus using TRIZOL reagent. cDNA was obtained using a Takara PrimeScript RT reagent kit according to the manufacturer's protocol. mRNA of NRF1, TFAM, Bax, Bcl-2, and caspase-3 was determined by the Takara TB Green™ Premix Ex Taq™ (Tli RNaseH Plus) kit (RR820A) according to the manufacturer's instructions with the ABI7500Real-Time PCR system (Bio-Rad, CA, USA). The PCR reaction system was 25 *μ*L (SYBR Premix Ex Taq II (Tli RNaseH Plus, 2x) 12.5 *μ*L, Primer F (10 *μ*M) 1 *μ*L, Primer R (10 *μ*M) 1 *μ*L, cDNA 2 *μ*L, and dH_2_O 8.5 *μ*L). The PCR conditions were as follows: 95°C for 30 seconds and 40 cycles of 95°C for 5 seconds, 60°C for 34 seconds, followed by a melting curve analysis (95°C for 15 seconds, 60°C for 1 minute, and then 95°C for 30 seconds and 60°C for 15 seconds). The sequences of the forward and reverse primers, purchased from Takara Biotechnology, used in this study are summarized in Table 1. Gene expression was normalized to *β*-actin and calculated by the 2-*ΔΔ*ct method. Primers were amplified with approximately equal efficiencies.

### 2.10. TUNEL Assay

Brain sections were deparaffinized in xylene and rehydrated via graded alcohols. Sections were hydrated and hydrolyzed by proteinase K working solution for 10 min. After being washed with PBS for 3 times, sections were treated with 20% normal bovine serum and incubated for 30 min. Then, the sections were treated with TdT buffer for 5 min and incubated in a humid chamber for 1 h after adding 50 *μ*L TdT reaction solution dropwise. Then, sections were kept warm in prewarmed washing buffer at 37°C for 30 min. After being washed with PBS for 3 times, sections were treated with DAB and incubated at 37°C for 5 min. TUNEL-positive cells were stained with a brown-yellow color and were observed under a light microscope (×400 magnification). Three nonoverlapping fields were randomly selected and photographed to record the total number of TUNEL-positive cells. TUNEL-positive cells (%) were calculated as follows: (positive staining cells (brown)/total cells) × 100%.

### 2.11. Statistical Analysis

Statistical analysis was performed using SPSS17.0 software. Results were reported as mean ± SEM. The significance of variables among groups was calculated by one-way ANOVA, followed by the Student-Newman-Keuls test and Dunnett's multiple comparison test. A *p* value of <0.05 was considered statistically significant.

## 3. Results

### 3.1. Animal Model

**Figure 2:**
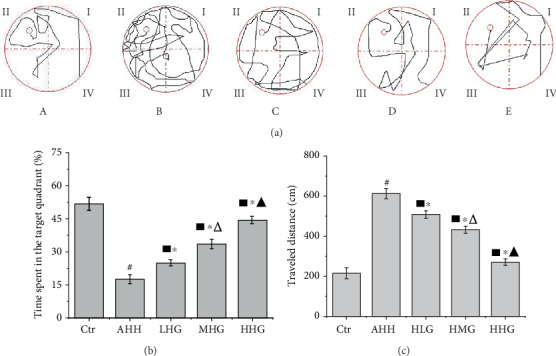
Crocin pretreatment ameliorated learning and memory impairment in acute high-altitude hypoxia rats (*n* = 12). Ctr: control group, with saline. AHH: rats exposed under hypoxia for 3 days at 4200 m. LHG, MHG, HHG: rats exposed under hypoxia for 3 days at 4200 m after pretreatment by 25 mg/kg/d, 50 mg/kg/d, and 100 mg/kg/d of crocin. (a) Characteristic swimming direction of the rats during the Morris water maze (MWM) test. (A) Ctr; (B) AHH; (C) LHG; (D) MHG; (E) HHG. (b) The percentage of time spent in the target quadrant. (c) Traveled distance. ^#^*p* < 0.05 versus Ctr, ^∗^*p* < 0.05 versus AHH, ^■^*p* < 0.05 versus Ctr, ^△^*p* < 0.05 versus HLG, and ^▲^*p* < 0.05 versus HMG.

We first performed the Morris water maze (MWM) test to evaluate the cognitive performance of different groups of rats (Figure 2). In the acute high-altitude hypoxia group (AHH), the traveled distance in the MWM test was significantly increased; the percentage of time in the target quadrant was significantly decreased (*p* < 0.05, vs. control group, Figure 2). The rat model of acute hypoxia-induced cognitive impairment was successfully established.

### 3.2. Animal Characteristics

There were no significant differences of body weight a in each group (*p* > 0.05, Table 2). The blood routine examination showed a significant increase in WBC in the AHH group when compared with that in the Ctr group (*p* < 0.05, Table 2). The MHG and HHG groups showed a significant decrease in WBC and increase in PLT when compared with the AHH group (*p* < 0.05, Table 2). However, RBC and PLT had no significant differences each group (*p* > 0.05, Table 2).

### 3.3. Crocin Pretreatment Improved Spatial Memory in Acute High-Altitude Hypoxia Rats

We found a significant increased percentage of time spent in the target quadrant and decreased traveled distance in the group pretreated with crocin compared with the AHH group (*p* < 0.05, Figures 2(a)–2(c)). Our results suggested that crocin pretreatment improved learning and memory performance in acute high-altitude hypoxia rats in a dose-dependent manner.

### 3.4. Effect of Crocin Pretreatment on Histopathological Changes of the Hippocampus in Acute High-Altitude Hypoxia Rats

**Figure 3:**
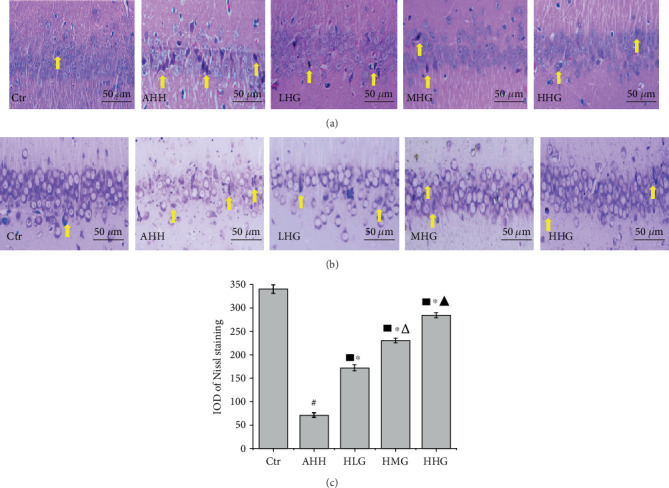
Crocin pretreatment improved the neuronal morphology of the hippocampus in rats under acute high-altitude hypoxia (*n* = 6, ×400 magnification, scale bar: 50 *μ*m). Ctr: control group, with saline. AHH: rats exposed under hypoxia for 3 days at 4200 m. LHG, MHG, HHG: rats exposed under hypoxia for 3 days at 4200 m after pretreatment by 25 mg/kg/d, 50 mg/kg/d, and 100 mg/kg/d of crocin. (a) HE-stained sections across the hippocampal CA1 region. (b, c) Nissl-stained sections across the hippocampal CA1 region. (↑ neurons shrunk and nuclear pyknosis). ^#^*p* < 0.05 versus Ctr, ^∗^*p* < 0.05 versus AHH, ^■^*p* < 0.05 versus Ctr, ^△^*p* < 0.05 versus HLG, and ^▲^*p* < 0.05 versus HMG.

The hippocampal neurons of the control group showed an ordered arrangement, normal structure, clear nuclei, and distinct nucleoli. The hippocampal neurons in the CA1 region showed a distinct and regular structure and were densely and clearly arranged in the control group (Figure 3(a)). However, the structure of the hippocampal neurons in the CA1 region in the AHH group was severely damaged compared with the control group. In the AHH group, the number of cells was reduced, the arrangement of the cells was chaotic, and the neurons were shrunk, nuclear pyknosis, and with an eosinophilic cytoplasm. Crocin pretreatment at all the doses alleviated the pathological damage of hippocampal neurons.

Neurons appeared in a dense arrangement, and the Nissl bodies were abundant in the CA1 region of the hippocampus of the control group (Figures 3(b) and 3(c)). The AHH group showed a reduced amount of Nissl bodies (71.01 ± 4.85) compared with the control group (340.29 ± 9.02), with a significantly decreased IOD value (*p* < 0.05). However, the IOD value was significantly increased in the crocin pretreatment groups at all the used concentrations compared with the AHH group (*p* < 0.05). Pretreatment of crocin significantly inhibited neuronal loss and prevented neuronal Nissl bodies' reduction in the hippocampus. The IOD value in the HHG group (284.23 ± 5.67) was significantly higher than that in the HLG (171.65 ± 6.59) and HMG (230.37 ± 4.76) groups (*p* < 0.05). Crocin pretreatment significantly ameliorated the structural damage of hippocampal cells in rat under acute high-altitude hypoxia exposure.

### 3.5. Crocin Pretreatment Improves the Expression of Mitochondrial Biosynthesis-Related Factors in Rat Hippocampal Cells under Acute High-Altitude Hypoxia

**Figure 4:**
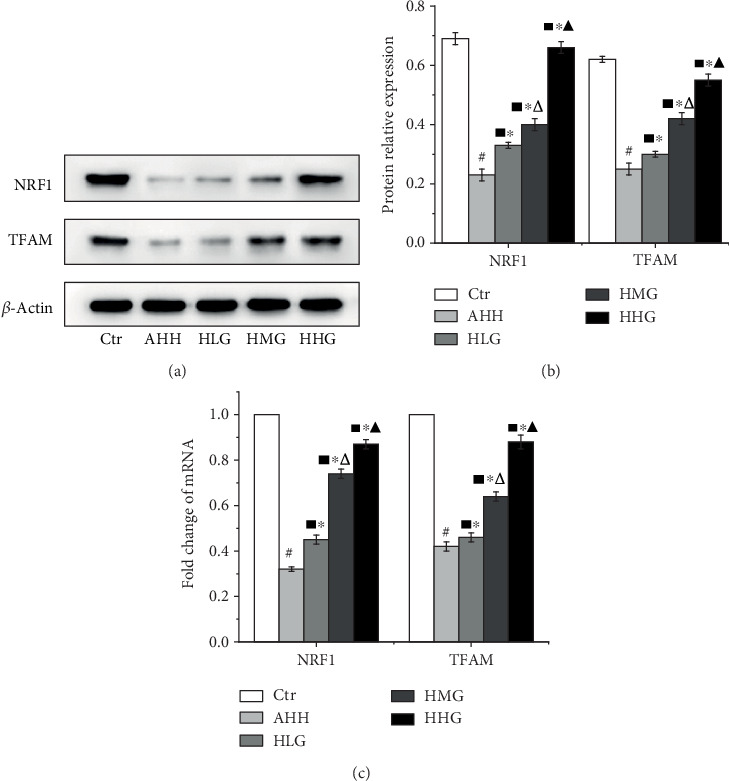
Crocin pretreatment improves the expression of mitochondrial biosynthesis-related factors in rat's hippocampal cells under acute high-altitude hypoxia. Ctr: control group, with saline. AHH: rats exposed under hypoxia for 3 days at 4200 m. LHG, MHG, HHG: rats exposed under hypoxia for 3 days at 4200 m after pretreatment by 25 mg/kg/d, 50 mg/kg/d, and 100 mg/kg/d of crocin). (a, b) Western blot detection of NRF1 and TFAM protein expression in the hippocampus (*n* = 12). (c) NRF1 and TFAM mRNA expression by qRT-PCR in the hippocampus (*n* = 12). ^#^*p* < 0.05 versus Ctr, ^∗^*p* < 0.05 versus AHH, ^■^*p* < 0.05 versus Ctr, ^△^*p* < 0.05 versus HLG, and ^▲^*p* < 0.05 versus HMG.

**Figure 5:**
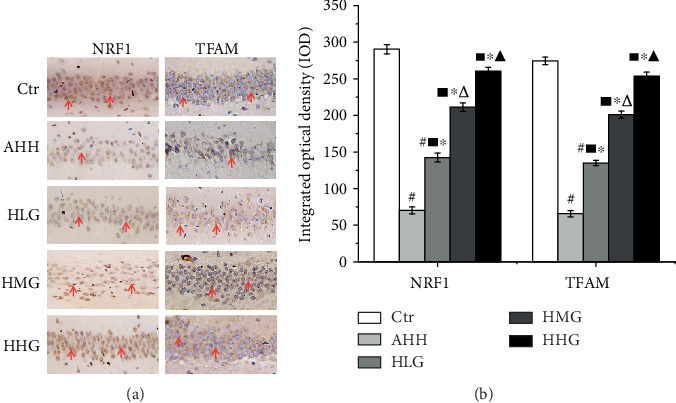
Crocin pretreatment improves the expression of NRF1 and TFAM in rat's hippocampal cells under acute high-altitude hypoxia. Ctr: control group, with saline. AHH: rats exposed under hypoxia for 3 days at 4200 m. LHG, MHG, HHG: rats exposed under hypoxia for 3 days at 4200 m after pretreatment by 25 mg/kg/d, 50 mg/kg/d, and 100 mg/kg/d of crocin. (a, b) Immunohistochemical staining of NRF1 and TFAM expression in the CA1 region of the hippocampus (*n* = 6). ^#^*p* < 0.05 versus Ctr, ^∗^*p* < 0.05 versus AHH, ^■^*p* < 0.05 versus Ctr, ^△^*p* < 0.05 versus HLG, and ^▲^*p* < 0.05 versus HMG.

Our previous studies show that in the AHH group, obvious injuries occurred, including a remarkable cell loss and karyopyknosis, swollen and decreased mitochondria, without cristae and with a fragmented double membrane structure. Crocin pretreatment significantly reduced injuries compared with the AHH group. In all crocin-treated groups, the number of mitochondria was significantly increased. The mitochondrial injury, including swelling, crest disappearance, and membrane structure damage, was ameliorated, suggesting a protection of the mitochondrial structure [19]. In this article, the results of real-time PCR and western blot showed that the level of NRF1 and TFAM in the AHH group was significantly decreased than that in the control group in rat's hippocampus (*p* < 0.05, Figures 4(a)–4(c)). In all crocin-pretreated groups, the level of NRF1 and TFAM was significantly increased than that of the AHH group (*p* < 0.05) in a dose-dependent manner. Meanwhile, we detected the expression of NRF1 and TFAM in the CA1 region of rat's hippocampus by the immunohistochemical test. The results showed that TFAM protein was expressed in cytoplasm and NRF1 protein was expressed in nucleus, showing a dark brown color in positive staining areas (Figures 5(a) and 5(b)). The IOD of NRF1 and TFAM in the AHH group was clearly decreased compared with the control group (*p* < 0.05). After treatment by crocin, the IOD of NRF1 and TFAM was also significantly increased compared with the AHH group (*p* < 0.05) in a dose-dependent manner.

### 3.6. Crocin Pretreatment Improved the Antioxidant Capacity in the Hippocampus in Acute High-Altitude Hypoxia Rats

**Figure 6:**
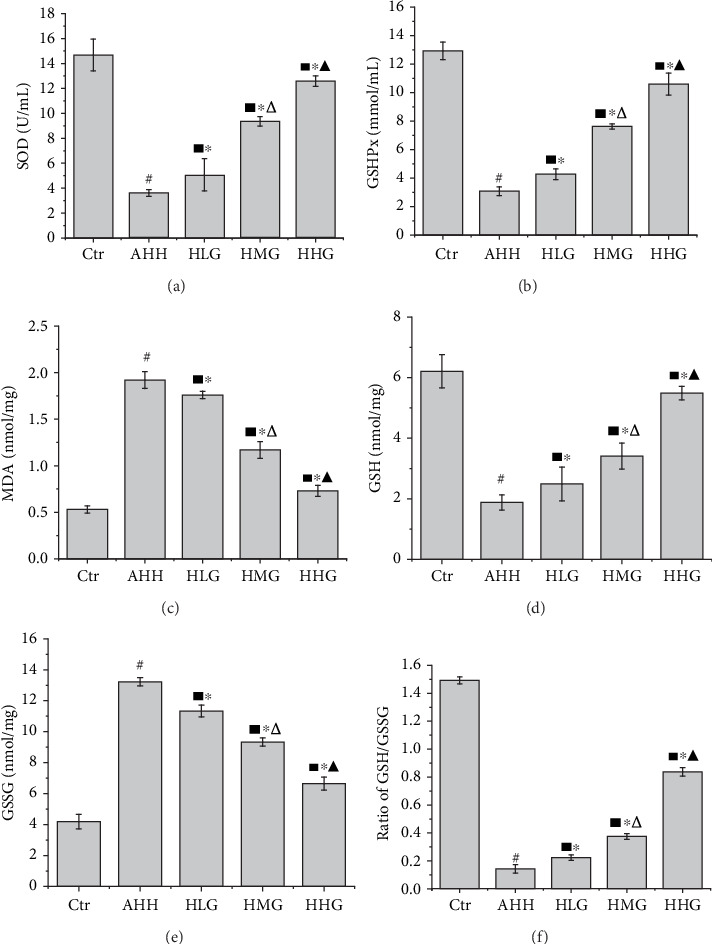
Crocin pretreatment improved the antioxidant capacity in the hippocampus of rats under acute high-altitude hypoxia (*n* = 12). Ctr: control group, with saline. AHH: rats exposed under hypoxia for 3 days at 4200 m. LHG, MHG, HHG: rats exposed under hypoxia for 3 days at 4200 m after pretreatment by 25 mg/kg/d, 50 mg/kg/d, and 100 mg/kg/d of crocin). (a) The levels of SOD. (b) The levels of GSHPx. (c) The levels of MDA. (d) The levels of GSH. (e) The levels of GSSG. (f) The GSH/GSSG ratio. ^#^*p* < 0.05 versus Ctr, ^∗^*p* < 0.05 versus AHH, ^■^*p* < 0.05 versus Ctr, ^△^*p* < 0.05 versus HLG, and ^▲^*p* < 0.05 versus HMG.

SOD (Figure 6(a)), GSHPx (Figure 6(b)), and GSH (Figure 6(d)) levels were significantly decreased; MDA (Figure 6(c)) and GSSG (Figure 6(e)) concentration was significantly increased in the AHH group; the GSH/GSSG ratio was also significantly reduced (*p* < 0.05, vs. control group). Pretreatment with crocin at all the concentrations significantly reduced the MDA and GSSG level and increased the level of SOD, GSHPx, and GSH, increasing the GSH/GSSG ratio at the same time compared with the AHH group (*p* < 0.05). So, crocin pretreatment significantly improved the antioxidant capacity of hippocampus in acute high-altitude hypoxia rats.

### 3.7. Crocin Pretreatment Protected Hippocampal Neurons by Inhibiting Neuronal Apoptosis in Acute High-Altitude Hypoxia Rats

**Figure 7:**
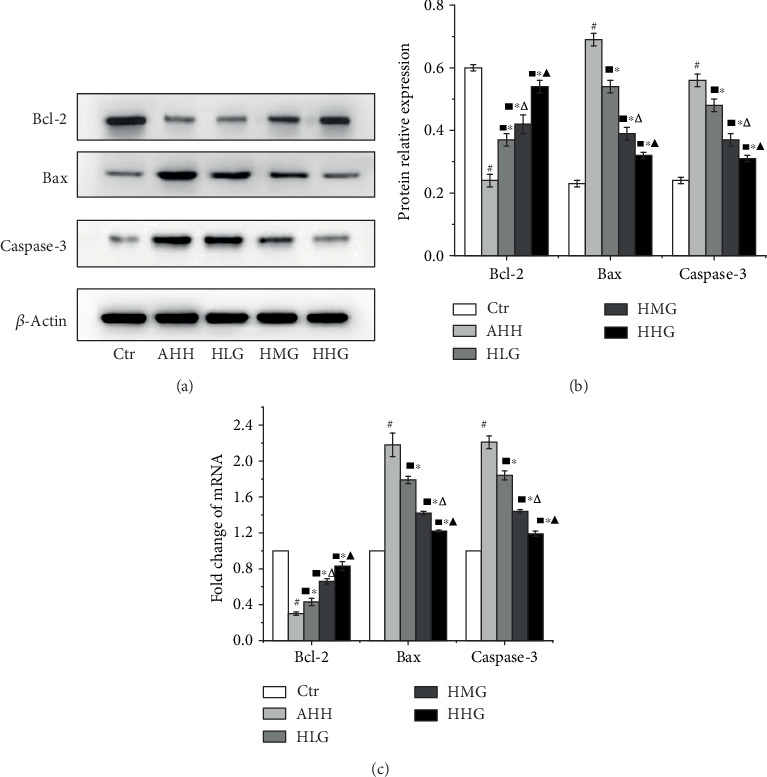
Crocin pretreatment decreased the Bcl-2, Bax, and caspase-3 expression in the hippocampus of rats under acute high-altitude hypoxia (*n* = 12). Ctr: control group, with saline. AHH: rats exposed under hypoxia for 3 days at 4200 m. LHG, MHG, HHG: rats exposed under hypoxia for 3 days at 4200 m after pretreatment by 25 mg/kg/d, 50 mg/kg/d, and 100 mg/kg/d of crocin. (a, b) Western blotting detection of Bcl-2, Bax, and caspase-3 protein expression in the hippocampus. (c) Bcl-2, Bax, and caspase-3 mRNA expression by qRT-PCR in the hippocampus. ^#^*p* < 0.05 versus Ctr, ^∗^*p* < 0.05 versus AHH, ^■^*p* < 0.05 versus Ctr, ^△^*p* < 0.05 versus HLG, and ^▲^*p* < 0.05 versus HMG.

**Figure 8:**
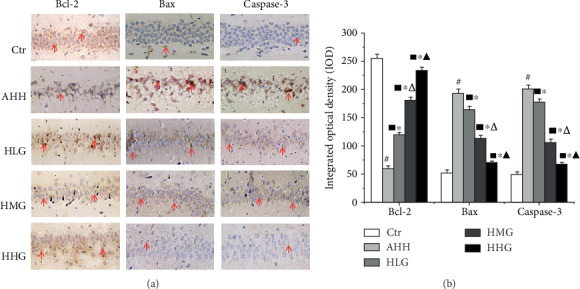
Crocin pretreatment decreased the Bcl-2, Bax, and caspase-3 expression in the CA1 region of hippocampal neurons under acute high-altitude hypoxia (*n* = 6). Ctr: control group, with saline. AHH: rats exposed under hypoxia for 3 days at 4200 m. LHG, MHG, HHG: rats exposed under hypoxia for 3 days at 4200 m after pretreatment by 25 mg/kg/d, 50 mg/kg/d, and 100 mg/kg/d of crocin. (a, b) Proteins were expressed in the cytoplasm, showing a dark brown color in positive staining areas. ^#^*p* < 0.05 versus Ctr, ^∗^*p* < 0.05 versus AHH, ^■^*p* < 0.05 versus Ctr, ^△^*p* < 0.05 versus HLG, and ^▲^*p* < 0.05 versus HMG.

We examined subsequently the expression of Bcl-2, Bax, and caspase-3 at mRNA and protein levels (Figures 7(a)–7(c)), and Bcl-2 (Figures 7(a)–7(c)) protein expression in the hippocampus of rats in the AHH group was significantly lower than that in the control group (*p* < 0.05), while Bax and caspase-3 were significantly higher than that in the control group (*p* < 0.05, Figures 7(a)–7(c)). Pretreatment with crocin at all the concentrations significantly increased the Bcl-2 proteins compared with the AHH group, while crocin decreased the level of Bax and caspase-3 protein expression (*p* < 0.05) in a dose-dependent manner. Meanwhile, we detected the expression of Bcl-2, Bax, and caspase-3 in hippocampal neurons in the CA1 area by the immunohistochemical test. Bcl-2, Bax, and caspase-3 (Figures 8(a) and 8(b)) proteins were expressed in the cytoplasm, showing a dark brown color in positive staining areas. The IOD of Bcl-2 was decreased, and the IOD of Bax and caspase-3 was increased significantly (*p* < 0.05, AHH group vs. control group). The IOD of Bcl-2 was also significantly increased in the crocin-pretreated groups (*p* < 0.05) compared with that in the AHH group, while IOD of Bax and caspase-3 was significantly decreased (*p* < 0.05) in a dose-dependent manner.

**Figure 9:**
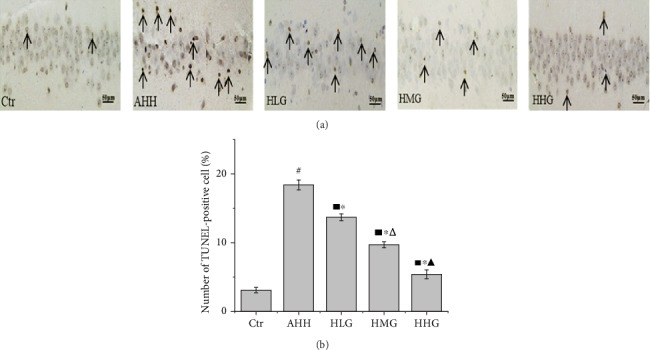
Crocin pretreatment inhibited neuronal apoptosis in the CA1 region of hippocampal neurons under acute high-altitude hypoxia (*n* = 6). Ctr: control group, with saline. AHH: rats exposed under hypoxia for 3 days at 4200 m. LHG, MHG, HHG: rats exposed under hypoxia for 3 days at 4200 m after pretreatment by 25 mg/kg/d, 50 mg/kg/d, and 100 mg/kg/d of crocin). (a, b) Quantification of TUNEL-positive cells in the hippocampus CA1 region. ^#^*p* < 0.05 versus Ctr, ^∗^*p* < 0.05 versus AHH, ^■^*p* < 0.05 versus Ctr, ^△^*p* < 0.05 versus HLG, and ^▲^*p* < 0.05 versus HMG.

As shown in the results of TUNEL staining, the number of TUNEL-positive cells in the hippocampal CA1 region of the AHH group was significantly increased compared with that of the control group (*p* < 0.05, Figure 9(a)). Pretreatment with crocin significantly reduced the number of positive cell (Figure 9(b)). Crocin upregulated the expression of Bcl-2, while downregulated the expression of caspase-3 and Bax in a dose-dependent manner, so as to inhibit the harmful effect of hypoxia on these proteins, suggesting that crocin protected hippocampal neurons from acute hypoxia injury by inhibiting neuron apoptosis.

## 4. Discussion

In our previous studies, we observed that crocin pretreatment improved cognitive function in rat under acute high-altitude hypoxia. However, the mechanism of crocin's brain protection remains unclear.

Crocin is the major component of saffron, with antioxidant and anti-inflammatory properties, and antiapoptotic, anticancer, hypolipidemic, neuroprotective, antiaging, and other broad pharmacological effects [21]. At high altitude, there is a decrease in the barometric pressure and a consequent reduction in the oxygen partial pressure (PO_2_), representing an extreme environmental condition [22]. Hypoxia at high altitude causes an imbalance of oxygenous availability to the tissues, causing severe physiological and psychological dysfunction in humans and other animals. Acute hypoxia severely impairs cognition and learning in humans [23]. Crocin mitigates malathion-induced neurological alterations and cognitive impairment by reducing oxidative stress and inflammatory reaction [24]. In addition, crocin improves cognitive performance and reduces hyperglycemia and oxidative stress in diabetic rats [25]. Our study found that crocin pretreatment could significantly improve learning and memory ability in rats induced by acute high-altitude hypoxia. The MWM test showed a significant decrease in traveled distance and increase percentage of time spent in the target quadrant in the crocin groups compared with the hypoxia group in a dose-dependent manner. Based on these observations, further experiments were conducted in order to clarify the protective mechanism of crocin pretreatment. The cells of the immune system are particularly sensitive to changes in oxidant stress because polyunsaturated fatty acids in their plasma membranes are highly susceptible to oxidative stress [26]. It was found that under the condition of 7576 and 5486.4 m hypobaric hypoxia, the circulating white blood cells (WBC) of rats increased significantly; therefore, it is speculated that hypoxia will cause oxidative stress, which will increase the number and activity of white blood cells [27]. Moreover, we also found that the number of white blood cells in hypoxic rats increased significantly, and crocin pretreatment could significantly decrease the number of white blood cells.

The hippocampus is an essential area in the brain for learning and memory, so the structure and function of hippocampal neurons are of great concern [28]. It was reported that the hippocampus was seriously damaged, and the number of pyramidal cells was decreased in rat under acute high-altitude hypoxia [29]. Moreover, exposure to hypoxia leads to chromatinic condensation and neurodegeneration in the hippocampus [30]. Our results showed that the neurons in the hippocampus of rats were disordered and deformed, and Nissl body was decreased significantly after 72 h rapidly of acute hypoxia exposure at high altitude.

Mitochondrial structural damage and dysfunction are the prominent pathological features in the brain under hypoxia [6]. In our previous study, the ultrastructural observation showed the presence of mitochondrial swelling and crista disappearance and a reduced number of mitochondria in the hippocampal neurons under acute hypoxia at high altitude; hippocampal neurons of crocin-pretreated groups were significantly protected from these injuries including cell loss and karyopyknosis [19].

SIRT1 plays a critical role in the regulation of various metabolic and pathophysiological processes, such as glycometabolism and lipid metabolism, inflammation, senescence, apoptosis, DNA damage repair, autophagy, oxidative stress, and cancer [31, 32]. Mitochondria enhance neuronal functionality and strengthen their resistance to stress, injury, and disease [33]. Research has discovered that long-term memory formation is mediated by SIRT, and enhancing the SIRT1 expression could resist the neurodegeneration in Huntington's disease (HD) mouse brain [34]. Lack of SIRT1 could damage cognitive function including imaginative memory, space learning, and recognition memory. In contrast, overexpression of SIRT1 could perform normal synaptic plasticity and memory capacity [35]. Moreover, SIRT1 is indispensable for synapse growth as well as memory formation and plasticity. Furthermore, SIRT1 could deacetylate PGC-1*α* to activate the PGC-1*α*-NRF-1-TFAM pathway. Overexpression of PGC-1*α* promotes synaptic differentiation, whereas its downregulation has the opposite effect causing neuropathology [35]. The study found that upregulation of the PGC-1*α*-NRF1-TFAM pathway promoted mitochondrial biogenesis, alleviated hippocampal neuronal injury, and improved spatial memory deficits in rats with hypoxia–ischemia [36]. Mitochondrial biogenesis and function require the coordinated transcription of nuclear and mitochondrial encoded genes and are mediated via transcriptional regulators that respond to extracellular and mitochondrial cues [37]. Therefore, promoting mitochondrial biogenesis plays an important role for neuronal function. Mitochondria are the major ROS producer and exert a crucial role within the cell-mediating processes including apoptosis, detoxification, and Ca^2+^ buffering [38]. This pivotal role makes mitochondria a potential target to treat a great variety of diseases.

Mitochondrial biogenesis is crucially important in modern neurochemistry because of the broad spectrum of human diseases arising from defects in mitochondrion and ROS homeostasis, energy production, and morphology. Meanwhile, the research found that the biogenesis of mitochondria could be pharmacologically controlled [39]. Several reports indicated that the SIRT1 and PGC-1*α* serve as master regulators of mitochondrial biogenesis and function by controlling gene expression [40], while SIRT1, PGC-1*α*, NRF1, and TFAM promoted the energy synthesis and survival rate of neurons [41]. SIRT1 affects mitochondrial biogenesis by regulating the transcriptional expression of PGC-1*α*, NRF1, and TFAM [42]. The SIRT1/PGC-1*α*/NRF1/TFAM is a central regulator of mitochondrial biosynthesis, and mitochondrial DNA is activated and upregulated by the NRF1 and TFAM [43]. NRF1 is a transcription factor that modulates genes controlling mitochondrial biogenesis and genes involved in diverse cellular functions [44]. According to a published report, upregulating the expression of the SIRT1/PGC-1*α*/NRF1/TFAM pathway could increase the expression of mtDNA and mitochondrial biogenesis. In this way, impaired mitochondria might be rescued by the activation of mitochondrial biogenesis or regeneration of new mitochondria [45]. The expression of mitochondrial biogenesis genes is known to be under the control of the PGC-1*α* and their activator SIRT1. The increase of PGC-1*α* activity could enhance the expression of NRF1 gene, while NRF1 could promote the expression of TFAM which directly stimulates mitochondrial DNA replication and transcription [46]. In our study, NRF1 and TFAM gene and protein expression in the hippocampus of rats were significantly decreased under acute high-altitude hypoxia. Pretreatment with crocin significantly increased the level of SIRT1, PGC-1*α*, NRF1, and TFAM and reduced the mitochondrial damage significantly, suggesting that crocin's protective effect was through increasing mitochondrial synthesis.

PGC-1*α* is one of regulators of mitochondrial biogenesis and function, and it plays a remarkable role in the resistance to oxidative stress and in the maintain of mitochondrial integrity [47]. Nuclear respiratory factor 1 (NRF1), perceived as a protein regulating genes controlling mitochondrial biogenesis, is now widely recognized as a multifunctional protein and as a key player in the transcriptional modulation of genes implicated in various cellular functions, including proliferation, and apoptosis [48]. It was reported that upregulating SIRT1, PGC-1*α*, NRF1, and TFAM reduced neuronal death by alleviating oxidative stress [49]. In addition, when neurons undergo oxidative stress challenge, increased PGC-1*α* regulated the cell response through upregulating the expression of antioxidative enzyme (including SOD) [50]. Increased oxidative stress represents an imbalance between intracellular production of free radicals and the cellular defense mechanisms. The sources of ROS are both extracellular (environment, drugs, or radiation) and intracellular (mitochondria or endoplasmic reticulum) [51]. Mitochondrial damage leading to high levels of ROS or MDA is considered to be one of the indicators of oxidative stress. On the other hand, enzymatic ROS scavenging mechanisms include SOD and glutathione peroxidase acts as the first line of defense against excess ROS [52]. Under hypobaric hypoxia, the antioxidant defense system such as GSH, GSHPx, SOD, and GSH/GSSG levels was significantly decreased in the hippocampus [53]. Crocin has been shown to be an antioxidant and neural protective agent. Crocin treatment has shown to counteract oxidative stress by reducing lipid peroxidation and improving the activity of antioxidant enzymes like SOD in some neurodegenerative disorder [54, 55]. These antioxidant effects of crocin were more effective than those of a-tocopherol at the same concentration on neuronally differentiated pheochromocytoma (PC-12) cells deprived of serum/glucose [56]. In a rat model of malathion-induced depressive-like behavior, crocin significantly decreased MDA levels and increased GSH levels in the hippocampus of rats compared with vitamin E [57]. Our study found that under the condition of acute hypoxia at high altitude, the hippocampus was in a state of high oxidative stress. The pretreatment of crocin played a good role in antioxidant stress; the protective effect of crocin was more obvious at higher doses especially at 100 mg/kg/d.

According to the published result, the activation of SIRT1 promotes the recovery of mitochondrial protein and function by increasing the biosynthesis of mitochondria and reduces neuronal apoptosis through the PGC-1*α* mitochondrial pathway [58]. PGC-1*α*, NRF1, and TFAM play an antiapoptotic role by decreasing Bax expression and increasing Bcl-2 expression through mitochondrial biosynthesis and antioxidant stress [59]. Exposure to hypoxia increased the number of TUNEL-positive cells along with the increase of caspase-3 expression in the CA1 region of the hippocampus [60]. Release of cytochrome c into the cytosol during hypoxic stress triggers apoptosis in neuronal cells [61]. Hypoxia increases the levels of caspase-3 and induces apoptosis in the hippocampal CA1 pyramidal neurons [62]. Moreover, SIRT1 significantly exerted an antiapoptotic effect by deacetylating lysine residue binding to protein kinase B and decreasing the activity of caspase-3, caspase-9, and related pathways [63]. In our study, crocin played a protective role in hippocampal neuron from oxidative stress damage under acute high-altitude hypoxia in a dose-dependent manner, ensuring neuronic viability and improving GSHPx, GSH, and SOD activity while reducing expression levels of MDA, GSSG, and ratio of GSH/GSSG. Crocin kept high expression of SIRT1, PGC-1*α*, NRF1, and Bcl-2 and kept a low expression of caspase-3 and Bax, suggesting that crocin protected hippocampal neurons against acute high-altitude hypoxia injury by inhibiting neuronal apoptosis and activating the SIRT1/PGC-1*α*/NRF1/TFAM signaling pathway.

## 5. Conclusion

Our results revealed that crocin significantly reduced hippocampal neuronic damage; improved the histomorphology of hippocampal neurons; markedly kept a high level of GSH, GSHPx, and SOD; reduced MDA and GSSG content; preserved a high SIRT1, PGC-1*α*, NRF1, TFAM, and Bcl-2 expression; decreased the level of Bax and caspase-3; and reduced the apoptotic rate. Taken together, our study found that crocin could attenuate the damage of hippocampal neurons induced by acute hypoxia by improving mitochondrial structure, reducing oxidative stress injury, and inhibiting neuronic apoptosis via the SIRT1/PGC-1*α*/NRF1/TFAM signaling pathway. Therefore, crocin might be considered as a potential therapeutic medicine to ameliorate cognitive impairment under acute high-altitude hypoxia.

## Figures and Tables

**Table 1:** List of primers used for qRT-PCR.

Gene symbol	GenBank accession	Sequence (5′ to 3′)	Product length (bp)
*NRF1*	NC_000007.14	F:TTACTCTGCTGTGGCTGATGG	143
		R:CCTCTGATGCTTGCGTCGTCT	

*TFAM*	NM_031326	F:TGAAGCTTGTAAATCAGGCTTGGA	146
		R:GAGATCACTTCGCCCAACTTCAG	

*Bcl-2*	NM_016993.1	F:GACTGAGTACCTGAACCGGCATC	135
		R:CTGAGCAGCGTCTTCAGAGACA	

*Bax*	NM_017059.2	F:TGGCGATGAACTGGACAACAA	65
		R:GGGAGTCTGTATCCACATCAGCA	

*Caspase-3*	NM_012922.2	F:GCAGCAGCCTCAAATTGTTGAC	144
		R:TGCTCCGGCTCAAACCATC	

*β-Actin*	NM_031144.2	F:CCTAAGGCCAACCGTGAAAA	103
		R:CAGAGGCATACAGGGACAACAC	

**Table 2:** Effects of crocin pretreatment on physiological indices of rats exposed to acute high hypoxic condition for 3 days (mean ± SEM, *n* = 12).

Group	BW (g)	WBC (10^9^/L)	RBC (10^12^/L)	PLT (10^9^/L)
Ctr	216.1 ± 6.2^#^	6.7 ± 0.3^∗^	8.5 ± 0.5^#^	771.5 ± 58^#^
AHH	214.8 ± 5.8	9.0 ± 0.5	8.8 ± 0.4	754.3 ± 47
HLG	214.4 ± 5.2^#^	8.1 ± 0.4	9.1 ± 0.6^#^	773.4 ± 68
HMG	217.4 ± 4.6^#^	6.6 ± 0.3^∗^	10.1 ± 0.5^#^	723.4 ± 75^#^
HHG	218.8 ± 6.3^#^	7.3 ± 0.4^∗^	9.9 ± 0.6^#^	751.6 ± 67^#^

Ctr: control group, with saline; AHH: rats exposed under hypoxia for 3 days at 4200 m; LHG, MHG, HHG: rats exposed under hypoxia for 3 days at 4200 m after pretreatment by 25 mg/kg/d, 50 mg/kg/d, and 100 mg/kg/d of crocin; BW: body weight; RBC: red blood cell; WBC: white blood cell; PLT: platelet. ^∗^*p* < 0.05 vs. AHH; ^#^*p* > 0.05 vs. AHH.

## Data Availability

The data used to support the findings of this study are available from the corresponding author upon request.
